# Novel Pathways for Injury from Offshore Oil Spills: Direct, Sublethal and Indirect Effects of the *Deepwater Horizon* Oil Spill on Pelagic *Sargassum* Communities

**DOI:** 10.1371/journal.pone.0074802

**Published:** 2013-09-25

**Authors:** Sean P. Powers, Frank J. Hernandez, Robert H. Condon, J. Marcus Drymon, Christopher M. Free

**Affiliations:** 1 Department of Marine Sciences, University of South Alabama, Mobile, Alabama, United States of America; 2 Dauphin Island Sea Lab, Dauphin Island, Alabama, United States of America; American University in Cairo, Egypt

## Abstract

The pelagic brown alga *Sargassum* forms an oasis of biodiversity and productivity in an otherwise featureless ocean surface. The vast pool of oil resulting from the *Deepwater Horizon* oil spill came into contact with a large portion of the Gulf of Mexico’s floating *Sargassum* mats. Aerial surveys performed during and after the oil spill show compelling evidence of loss and subsequent recovery of *Sargassum*. Expanding on the trends observed in the aerial surveys, we conducted a series of mesocosm experiments to test the effect of oil and dispersants on the vertical position and weight of the *Sargassum* complex (*Sargassum natans* and *S. fluitans*), as well as on the dissolved oxygen concentrations surrounding the algae. Dispersant and dispersed-oil had significant effects on the vertical position of both species of *Sargassum* over a period of 72 hours. Similarly, dissolved oxygen concentrations were lowest in dispersant and dispersed-oil treatments, respectively. Cumulatively, our findings suggest three pathways for oil-spill related injury: (1) *Sargassum* accumulated oil on the surface exposing animals to high concentrations of contaminants; (2) application of dispersant sank *Sargassum*, thus removing the habitat and potentially transporting oil and dispersant vertically; and (3) low oxygen surrounded the habitat potentially stressing animals that reside in the alga. These pathways represent direct, sublethal, and indirect effects of oil and dispersant release that minimize the ecosystem services provided by floating *Sargassum* – the latter two effects are rarely considered in assessing impacts of oil spills or response procedures.

## Introduction

Given the vast literature demonstrating direct negative impacts of oil spills, understanding how response activities mitigate or exacerbate the impacts of an oil spill is of fundamental importance to understanding the ecological effects of the *Deepwater Horizon* oil spill (DWH) and most importantly, developing appropriate, multidimensional policy for future responses to oil spills. One of the most contentious response activities during the DWH was the use of dispersants [1]. Dispersants are typically used to reduce the impact of oil on shorelines, reduce impacts of oil on surface dwelling birds and mammals, and promote the biodegradation of oil in the water column [2]. In contrast to the deep-water injection of dispersant, the application of dispersants to surface oil was a traditional response, although the sheer magnitude of dispersant application (3.7 million L [3]) was likely never envisioned by ecosystem managers (officially termed an atypical response activity). While surface application appeared to have successfully dispersed a large fraction of the oil throughout the water column, it also transformed the traditional 2-D footprint of a surface oil spill into a dramatically more complex 3-D problem [1]. One key oceanic habitat affected by the DWH was floating *Sargassum* mats (*Sargassum natans* and *S. fluitans)*. Given the extensive spatial and temporal scope of the DWH, the fate of *Sargassum* following the DWH in the Gulf of Mexico (GOM), which contains the second most productive *Sargassum* system in the world [4], [5], illustrates the profound ecological and socioeconomic tradeoffs that must be considered collectively and more fully when responding to oil spills.

*Sargassum* is a pelagic brown algae that represents an oasis of structure in the open ocean and supports a large and diverse assemblage of marine turtles [6], fish [7], [8], [9] and invertebrates [10]. In the northcentral GOM, *Sargassum* is composed almost exclusively of two species, *Sargassum natans* and *S. fluitans* [11]). Unlike other species of *Sargassum*, *S. natans* and *S. fluitans* are holopelagic, and are typically considered as a single complex (pelagic *Sargassum*, [12]). Pelagic *Sargassum* is a ubiquitous feature of the northcentral GOM where it occurs in three configurations depending on meteorological and *in situ* oceanic conditions: scattered clumps (during high winds), small and meso scale (1 m–10 km’s) convergence lines, and larger circular mats [13], [14]. Given its pelagic and ephemeral nature, systematic surveys of *Sargassum* abundance in the Gulf of Mexico are uncommon; thus we know of little historical abundance and distribution of this habitat.

The loss and degradation of another structurally complex brown algae *Fucus* in Prince William Sound, Alaska following the *Exxon Valdez* oil spill was responsible for a myriad of direct and indirect impacts on the food web, many of which persisted for years [15]. *Fucus* fulfills ecological functions similar to those of *Sargassum*; hence, we expect the consequences of any degradation of *Sargassum* to follow the responses documented for the loss of these brown algae. However, in contrast to *Fucus*, which supports a largely benthic and demersal community, *Sargassum* supports a pelagic ecosystem. Whereas *Fucus* represents one of a multitude of nearshore structurally complex habitats available for fish and invertebrates, *Sargassum* fulfills a unique position in the open ocean as the only naturally occurring biogenic habitat.

To quantify the potential impact of floating oil and dispersant application on pelagic *Sargassum* we first documented the distribution of *Sargassum* during and after the DWH in 2010–2012. Because of the clear overlap between *Sargassum* and oil as well as dispersant recorded in 2010, we then investigated the potential effects of *Sargassum* exposure to oil and dispersant via replicated mesocosm experiments.

## Methods

*Sargassum* for mesocosm experiments was collected in the offshore waters of the Gulf of Mexico. No specific permissions were required for this activity. Fish and invertebrates residing within the *Sargassum* were removed and returned to the water. None of these fish or invertebrates were endangered or protected, and no animals were killed specifically for this study.

### Aerial Surveys of *Sargassum* Distribution

**Figure 1:**
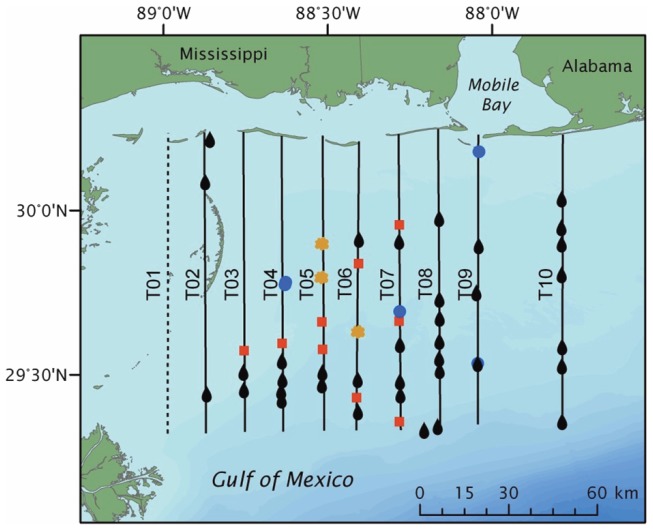
Results of an aerial survey documenting the co-occurrence of oil and *Sargassum,* June 2010. Results of one (6/16/2010) of 8 aerial surveys conducted during the summer of 2010 documenting oil presence (indicated by oil droplets), *Sargassum* (yellow floral symbols), dolphins (blue circles) and fish schools (red squares).

*Sargassum* can be patchily distributed in small clumps, or aggregated into large mats by Langmuir circulations or convergence along frontal zones. Strip-transect aerial surveys are commonly used to assess the abundance of surface-occurring marine fauna [16], [17]. We used a similar approach to locate *Sargassum* patches and convergence lines to estimate the quantity and distribution of *Sargassum* in relation to oil and dispersant application. Ten equally spaced transects were established within the area (the 9^th^ transect in the eastern section was dropped during the project due to time constraints). Each transect started at the shoreline and extended perpendicularly to approximately 100 km offshore (Figure 1). Ten aerial surveys (5/28, 6/4, 6/9, 6/16, 6/23, 7/14, 7/21, 8/18, 9/8, 10/20) were conducted after the DWH in 2010. Follow up flights were also flown in 2011 (7 flights, 7/22, 7/26, 8/17, 9/9, 9/15, 9/21, 9/28) and 2012 (2 flights, 5/16, 7/5). A twin prop Cessna (or similar) aircraft was used for each flight and flown between 215–300 m elevation depending on ceiling restrictions imposed by the unified command and Federal Aviation Administration (FAA) during the DWH explosion. To ensure adequate detectability of *Sargassum*, all surveys were conducted on days where sea state was equal to or less than 4 on the Beaufort wind force scale. Two observers were seated in the rear of the aircraft and recorded the latitude and longitude (via GPS) of targets (*Sargassum* mats or convergence lines, whales, turtles, dolphins, fish schools, etc.) within 85**°** under the plane wing. The altitude for each aerial survey varied according to changing FAA restrictions; therefore, the detectable area ranged from 2.44–3.48 km, depending on the flight altitude.

To examine temporal trends in abundance, observations from the aerial survey were used to create an index of *Sargassum* abundance. As is the case with many fishery-independent datasets, the aerial survey data had a high proportion of zero values (i.e. instances where no *Sargassum* was observed). As a result, we used a delta-lognormal approach [18], [19] that combines separate generalized linear models (GLM) of the probability of a non-zero observation (number of transects with positive sightings/total number of transects) and the observation rate along positive transects to construct a single standardized index of abundance, defined as:
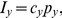
(1)where *c_y_* is the estimate of mean observations for positive observations for year *y*, and *p_y_* is the estimate of mean probability of occurrence during year *y*. Data used to estimate abundance for positive catches (*c*) and probability of occurrence (*p*) were assumed to have a lognormal distribution and a binomial distribution, respectively, and modeled using the following equations:
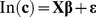
(2)and
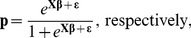
(3)where c is a vector of the positive observation data, p is a vector of the presence/absence data, X is the design matrix for main effects, β is the parameter vector for main effects, and ε is a vector of independent normally distributed errors with expectation zero and variance σ2. Coefficients of variation for the index were estimated using a jackknife routine. Year and season were used as covariates to control for seasonal effects on observation rates. Parameterization of each model was accomplished using R version 2.15.

### Mesoscom Experiments

To complement our aerial survey data, mesocosm experiments were conducted to explore oil spill related effects on *Sargassum*. *Sargassum* was collected in September 2013 at three locations south of Dauphin Island. Location 1 was 29° 27.095′ N, 87° 46.518′ W, location 2 was 29° 48.442′ N, 87° 56.302′ W, and location 3 was 29° 43.246′ N, 88° 00.528′ W. At these locations, *Sargassum* was removed from the water using large dip nets, shook to remove fish and invertebrates, and placed in large coolers. Epiphytic algae were not removed from the *Sargassum* prior to the start of experiments.

**Figure 2:**
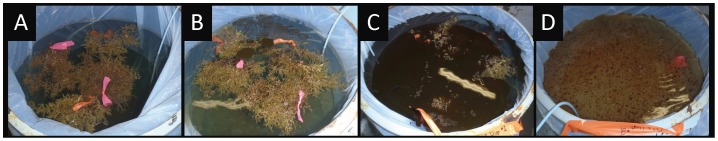
Evolution of experimental treatments. Example of mesocosms (A) prior to addition of oil, (B) following addition of 100 ml of Louisiana sweet crude oil, (C) five minutes after application of Corexit 9500 A dispersant, and (D) 12 hours after dispersant application.

We conducted two sets of experiments to determine the effects of oil and dispersant on floating *Sargassum*. For the first experiment, treatments were conducted and run under static conditions. The second set of experiments were run in an identical manner with the exception that air was bubbled slowly into the bottom of the tank to prevent low oxygen conditions from forming. Both sets of experiments were designed to test the hypothesis that exposure to moderate levels of oil or dispersant caused *Sargassum* to sink from its surficial habitat. In both experiments, four treatments were tested: 100 ml of LA sweet crude oil, 5 ml of Corexit 9500 A oil dispersant, dispersed-oil (dispersant+oil, 5 and 100 ml respectively), and a control (seawater). We chose 100 ml of crude oil after conducting preliminary trials where oil was released in varying concentrations on the surface. Based on visual examination, 100 ml of oil created distinct oil pockets that covered approximately 10% of the surface area – a realistic although conservative amount based on the authors’ on-water experience during the spill. MC 252 sweet crude oil was provided by BP Exploration and Production under agreement COC 20110512-HP1-053. Corexit 9500 A dispersant was supplied by Nalco. At the beginning of the trials, treatments (oil, dispersant, dispersed-oil and control) were measured with graduated cylinders and added to circular mesocosms (0.6 m diameter × 0.8 m depth) that contained 220 L of seawater (20–24 psu and 27–28°C) and were lined with Teflon bags (Figure 2). Four pre-weighed *Sargassum* colonies (2 *S. natans* and 2 *S.* *fluitans*) were then placed into the mesocosms. Both species of *Sargassum* co-occur in mats in the GOM and North Atlantic and we tracked the response of each species separately in case species-specific differences occurred. Four mesocosms, one for each of the four treatments, were placed in much larger circular seawater tanks (5000 L, 2 m tall filled to a level of 0.7 m) to maintain water temperature (+/−1°C). All treatments were replicated three times by placing 220 L circular mesocosms in three separate seawater tanks.

Vertical position of *Sargassum*, weight of *Sargassum* and dissolved oxygen concentrations were measured during both static and aerated experiments. During the experiments vertical position relative to the water surface of each of four colonies (2 *S. natans* and 2 *S*. *fluitans*) was measured every 24 hours for 72 hours. We chose 72 hours because our field observations during the oil spill suggested that this was a reasonable duration for oil and *Sargassum* to remain in close contact in surface waters. The four colonies used in each mesocosm (2 *S. fluitans* and 2 *S. natans*) were weighed and individually photographed at the beginning and end of each trial. All colonies had a small piece of fluorescent flagging tape (for ID) attached to the base of the colony. Colonies were photographed with their ID code showing. Prior to weighing, each colony was spun in a common household salad spinner for ten revolutions to remove excess water. Upon retrieval, colonies were spun again (in washed and de-oiled spinners) and weighed on a digital scale (+/−0.01 g). All material was wrapped in aluminum foil and frozen. *Sargassum* was weighed before and after the trials were completed and all trials were run for 72 hours. We monitored surface and bottom dissolved oxygen concentrations throughout both static and aerated experiments. Dissolved oxygen was monitored with a handheld Yellow Springs Instrument (YSI model #2030) dissolved oxygen meter.

A series of Analysis of Variance (ANOVAs) were used to test the response of *Sargassum* vertical position and weight change as well as dissolved oxygen concentrations in our experiments. We tested the hypothesis that the percent of colonies at the surface varied among treatments. Specifically, we used a repeated measures ANOVA with time (0, 24, 48 and 72 hour intervals) as a repeated measure and treatment (oil, dispersant, dispersed-oil and control) as an independent variable. The dependent variable in our analyses was the percent of *Sargassum* colonies in surface water. Finally, to examine the patterns in dissolved oxygen concentration, we performed a repeated measures ANOVA testing the effect of time (repeated measures 0, 24, 48, and 72 hours) and treatment (oil, dispersant, dispersed-oil, and control). Separate ANOVAs were performed for the static and aerated experiments. For all ANOVAs, no transformations of data were necessary to meet the requirements of homogeneity of variance (Cochran’s C test) or normality (K-S test).

## Results

### Aerial Surveys of *Sargassum* Distribution

**Figure 3:**
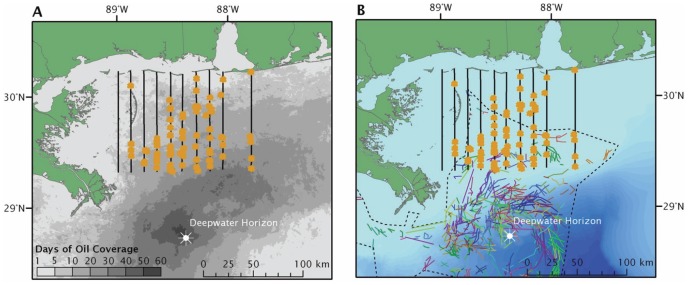
*Sargassum* targets in relation to oil and dispersant. Locations of *Sargassum* targets (yellow dots) identified along aerial flight transects (black lines) in 2010 relative to days of oil coverage (A) and application of dispersants (B). Transect 1, the dotted transect, was not flown in 2010 due to spill-related flight restrictions. In A, cumulative surface oiled was calculated through the analysis of NESDIS satellite imagery [*]. In B, the colored lines represent daily aerial spraying tracks and the dashed polygon is the full envelope of the effective area [2]. [*] Cumulative surface oiling of the Deepwater Horizon oil spill (2010) The Nature Conservancy, Coastal Resilience Gulf of Mexico Project (http://coastalresilience.org/gulfmex). Accessed November 7, 2012.

**Figure 4:**
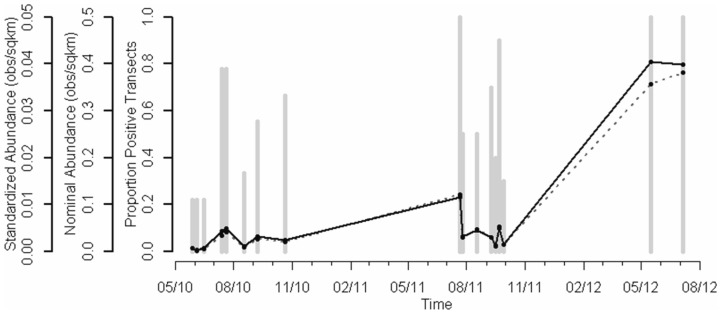
Index of relative *Sargassum* abundance. Standardized (bold line) number of *Sargassum* targets based on a delta-log normal abundance index. Positive transect lines are shown in gray bars, and the nominal number of targets per square km are shown with a dashed line.

At the onset of the oil spill, we began flying aerial transects to quantify overlap between *Sargassum* and surface oil. Ten aerial surveys each covering ∼3100 km^2^ of ocean surface from the panhandle of FL to the Chandeleur Islands, LA were completed in 2010 and documented extensive co-occurrence of oil and *Sargassum* (Figure 3A). Our survey grid was also within a larger area that was actively sprayed with the dispersant Corexit 9500 A (Figure 3B); thus we documented that *Sargassum* was exposed to both oil and dispersants. Follow-up aerial surveys in 2011 and 2012 (n = 9) documented a four-fold increase in *Sargassum* abundance since the DWH (Figure 4).

### Experiment 1: Static Conditions

**Figure 5:**
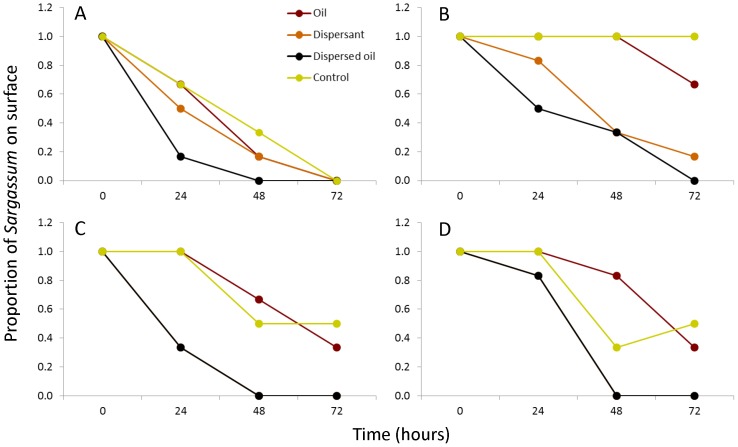
Percentage of surface *Sargassum* as a function of treatment. Percent of *Sargassum (S. natans and S. fluitans)* colonies remaining on surface for four treatments: oil, dispersant, dispersed-oil and control under both static and aerated conditions. Panels are as follows: static, *S. natans* (A), static, *S. fluitans* (B), aerated, *S. natans* (C), aerated, *S. fluitans* (D). In panels C and D, the dispersant and dispersed-oil treatment data are identical.

The proportion of *Sargassum* in surface water differed by oiling/dispersant treatments as well as over the course of the experiment as indicated by the significant treatment × time interaction in our ANOVA (Table 1, Figure 5A and B). This trend was most pronounced for *S. natans*, where all treatments started sinking after 24 hours. *Sargassum* in the control treatment sank slowest, followed by oil, dispersant, and dispersed-oil treatments (Figure 5A). An identical, yet less dramatic pattern was observed for *S. fluitans*, where all *Sargassum* in the control and oiled treatments remained at the surface through 48 hours (Figure 5B). Notably, for both *S. natans* and *S. fluitans*, no colonies in the dispersed-oil treatment were at the surface after 72 hours (Figure 5A and B).

**Table 1:** Test for between and within subject effects in repeated measures ANOVA with treatment (oil, dispersant, dispersed-oil and control) as fixed factors and time (0, 24, 48, and 72) as a repeated measures on the proportion of *S. fluitans* and *S. natans* colonies on the surface.

Between subject effects					
Source	DF	SS	MS	F	Pr>F
Treatment	3	2.714	0.905	13.838	<0.001
Error	20	1.307	0.065		
**Within subject effects**					
**Source**	**DF**	**SS**	**MS**	**F**	**Pr>F**
Time	3	8.807	2.936	92.654	<0.001
Treatment*Time	9	1.229	0.137	4.311	0.001
Error	60	1.901	0.032		

### Experiment 2: Aerated Conditions

Trends in the aerated experiments were similar to those observed during the static experiments. In the control treatment, 100% of colonies of both *S. natans* and *S. fluitans* were at the surface after 24 hours, after which time they began to sink. In the oil treatment, 67% of *S. natans* and 83% of *S. fluitans* were at the surface after 48 hours, and at 72 hours, only 33% of colonies of both species remained at the surface. For both *Sargassum* species in the dispersant and dispersed-oil treatments, no colonies were at the surface by 48 hours (Figure 5C and D).

### Dissolved Oxygen

**Figure 6:**
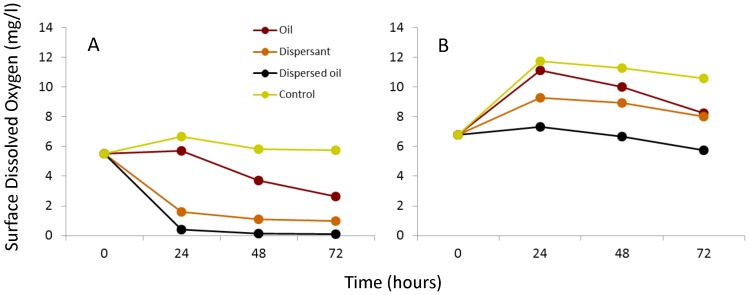
Surface water dissolved oxygen as a function of treatment. Dissolved oxygen concentrations in surface water for four treatments: oil, dispersant, dispersed-oil and control under static (A) and aerated (B) conditions.

A significant interaction was detected in the repeated measures ANOVAs testing the effect of time (repeated measures 0, 24, 48, and 72 hours) and treatment (oil, dispersant, dispersed-oil, and control) under both static (Table 2) and aerated (Table 3) conditions. Oxygen concentrations were significantly different in each treatment for both static and aerated conditions (dispersed-oil<dispersant<oil<control) (Figure 6). Under static conditions hypoxic and anoxic conditions formed within 24 hours under dispersed-oil and dispersant only treatments (Figure 6A). During the aerated experiments, the same ordering of treatment effects was seen (dispersed-oil<dispersant<oil<control), but with higher overall values (all >6.0 mg/L, Figure 6B).

**Table 2:** Test for between and within subject effects in repeated measures ANOVA with treatment (oil, dispersant, dispersed-oil and control) as a fixed factor on surface dissolved oxygen concentrations in the first experiment (static conditions).

Between subject effects					
Source	DF	SS	MS	F	Pr>F
Treatment	3	143.292	47.292	100.409	<0.001
Error	8	3.806	0.476		
**Within subject effects**					
**Source**	**DF**	**SS**	**MS**	**F**	**Pr>F**
Repetition	3	128.138	42.713	378.399	<0.001
Treatment*Repetition	9	56.688	6.299	55.801	<0.001
Error	24	2.709	0.133		

**Table 3:** Test for between and within subject effects in repeated measures ANOVA with treatment (oil, dispersant, dispersed-oil and control) as a fixed factor on surface dissolved oxygen concentrations in the second experiment (aerated conditions).

Between subject effects					
Source	DF	SS	MS	F	Pr>F
Treatment	3	77.741	25.914	15.168	0.001
Error	8	13.667	1.708		
**Within subject effects**					
**Source**	**DF**	**SS**	**MS**	**F**	**Pr>F**
Repetition	3	67.295	22.432	61.035	<0.001
Treatment*Repetition	9	27.779	3.087	8.398	<0.001
Error	24	8.821	0.368		

## Discussion

Aerial surveys of *Sargassum* distribution documented exposure to oil and dispersants and subsequent increase of the alga in the years following the oil spill. The lack of pre-spill baseline data on *Sargassum* abundance renders it impossible to ascribe the higher abundance of *Sargassum* contacts in 2011 and 2012 to an oil spill affect; however, the pattern is suggestive of a potential response to the unique conditions in 2010. Our experiments demonstrated that oil and dispersant had significant effects on the vertical position of *Sargassum* and thus offer a potential explanation for the dramatic trend seen in our aerial survey data. Both experiments 1 (static) and 2 (aeration) demonstrated significant effects of dispersant on the vertical position of *Sargassum*. In both the dispersant only and dispersed-oil treatments, all *Sargassum* sank to the bottom within 24–48 hours. In contrast, *Sargassum* in the oil only and control treatments remained at the surface of the tank for the majority of the experiment. The effect was more pronounced for *S. fluitans* than *S. natans*, where the latter tended to sink towards the end of the experiments in the control and oil treatment. These findings support a body of literature documenting increased toxicity of chemically-dispersed compared to physically-dispersed oil (reviewed in [2]).In the GOM, *S. natans* and *S. fluitans* generally co-occur within neustonic mats and a mixed colony is likely buoyed by the broader leaf species *S. fluitans*. Thus, our experiments demonstrate two potential pathways for injury to *Sargassum* and its associated fauna. First, *Sargassum* mixing with oil and remaining on the surface exposes numerous invertebrates, fish [20], and sea turtles [6], which are attracted to the floating *Sargassum*, to elevated concentrations of oil. Additionally, contaminated *Sargassum* remaining on the ocean surface serves as a horizontal export mechanism, potentially extending the impact of the spill via the “*Sargassum* conveyor belt” from the central GOM to the North Atlantic Ocean [5]. *Sargassum* maintains it surface position via berry-like vesicles whose gas composition is maintained through diffusion with surrounding waters [21]. Once *Sargassum* sinks, its value as refuge for neustonic fauna disappears. Reemergence on the surface depends on the ability of *Sargassum* to “refill” its vesicles and the sinking depth [22]; however, most *Sargassum* that sinks will not return to the surface. In this second pathway, vertical sinking exposes mesopelagic and benthic fauna to oil and dispersant trapped in the *Sargassum*, and may represent a significant labile carbon source for microbial communities that can consume dispersed oil and dead plant material. The loss of surface refuge is further exacerbated when one considers the lost production by healthy *Sargassum*, which propagates via fragmentation and growth. Assuming a 1-year life span and growth rate of 0.04 d^−1^ [23], a km^2^ of lost *Sargassum* would translate to a total loss of 25 km^2^ of *Sargassum* per year. When one considers density estimates of sea turtles per km^2^ range from 0.9–6.9 post hatchlings and 4.8–7.2 juveniles [17], the potential injury from habitat loss could easily contribute to a year-class failure for some higher order consumers.

A third mechanism for injury is also evident in the dissolved oxygen dynamics under static conditions in our first experiment. The addition of dispersant to our mesocosms rapidly depleted oxygen with the greatest oxygen consumption observed in the dispersed-oil and dispersant only treatments, likely caused by increased microbial respiration [24]. Within 12 hours, hypoxic (dissolved oxygen <2 mg/L) conditions formed in the two treatments with dispersant and from 24–36 hours anoxia (0 mg/L) had developed in the dispersed-oil treatment. Dissolved oxygen conditions were higher in the oil only (>3 mg/L) and control (>5 mg/L) treatments (p<0.05 for post hoc contrast). A similar ranking of treatments was seen under aeration conditions (dispersed-oil<dispersant<oil<control) although aeration prevented the formation of hypoxic conditions. Negative effects (increased mortality, increased physiological stress, and decreased fitness) are well documented under episodic or chronic low oxygen conditions in marine waters [25], [26]. If winds are weak, as is the case when large *Sargassum* mats form [13], surface mixing would be reduced in the GOM and more conducive to the formation of water masses with low oxygen surrounding oil and dispersant mixtures. Surface water hypoxia/anoxia would be accentuated under oiled conditions because surface slicks form a physical barrier that resists wind stress leading to less mixing of the water column and retarding gas exchange from the ocean to atmosphere [27]. Under these conditions, the presence of low oxygen waters represents a major indirect effect that could lead to injury.

Our results illustrate the potential for direct, sublethal, and indirect effects as pathways for injury from oil spills and the application of dispersants with major implications for the management of ecosystem resources and services. *Sargassum* remained alive in our trials; therefore, traditional LC_50_ survival tests or EC_10_ assessments would have failed to detect a negative effect of oil and dispersant exposure. In fact, no significant change in weight was recorded during the experiment beyond a trend of heavier weight in the oil treatment, which is likely explained by fouling of plant material with oil. The sublethal effect of *Sargassum*’s loss of buoyancy and resulting sinking in the presence of dispersants means that *Sargassum* would have eventually died and immediately removed most of its ecological and socioeconomic benefits. The loss and degradation of structurally complex brown algae (*Fucus*) in Prince William Sound, AK following the *Exxon Valdez* oil spill resulted in ecosystem wide effects [15]. Interestingly, the loss of *Fucus* was also primarily a function of response activities (high pressure washing denuded nearshore rocky areas [28]).

As demand for fossil fuels remains high, so does the potential for oil spills. Response activities are a necessary component of mitigating the impacts of such spills. Although the intentions behind all response activities are good, the effects of these activities may further exacerbate injury. In the GOM, dispersants were applied to seven oil spills between 1990 and 1998; in each instance, their application was deemed successful to some extent [29]. In the case of the DWH, the efficacy of spraying vast quantities of Corexit 9500 A to disperse surface oil remains to be examined; however, this evaluation must include a careful consideration of the associated tradeoffs. For *Sargassum* in the GOM, contact with dispersant may result in loss of this unique habitat, while increasing exposure at depth to mesopelagic and benthic communities, illustrating the complex scenarios that must be considered. Additionally, *Sargassum* in the GOM and North Atlantic Ocean can account for as much as 60% of primary production in the oligotrophic expanses [30]. The loss of such high productivity in oligotrophic areas is a significant concern for the ecosystem. With more offshore drilling operations likely in regions that support *Sargassum* communities, understanding the ecological risks is a critical necessity. Full consideration of the tradeoffs associated with dispersants and oil spills are required for effective management and policy implementation. Here, we demonstrate novel and important pathways currently not considered during the DWH.
